# *β*-Carboline Silver Compound Binding Studies with Human Serum Albumin: A Comprehensive Multispectroscopic Analysis and Molecular Modeling Study

**DOI:** 10.1155/2018/9782419

**Published:** 2018-03-25

**Authors:** Ali Alsalme, Rais Ahmad Khan, Arwa M. Alkathiri, Mohd. Sajid Ali, Sartaj Tabassum, Mohammed Jaafar, Hamad A. Al-Lohedan

**Affiliations:** ^1^Department of Chemistry, College of Science, King Saud University, P.O. Box 2455, Riyadh 11451, Saudi Arabia; ^2^Surfactant Research Chair, Department of Chemistry, College of Science, King Saud University, P.O. Box 2455, Riyadh 11451, Saudi Arabia

## Abstract

*β*-Carbolines (*β*Cs) belong to the naturally occurring alkaloid family, derived from 9H-pyrido[3,4-b]indole, also known as norharmane (Hnor). Knowing the importance of the *β*Cs alkaloid family in biological processes, a comprehensive binding study is reported of four Ag(I) compounds containing the ligand Hnor and having different counteranions, namely, NO_3_^−^, ClO_4_^−^, BF_4_^−^, and PF_6_^−^, with human serum albumin (HSA) as a model protein. Different approaches like UV-visible, fluorescence spectroscopy, circular dichroism (CD), and molecular docking studies have been used for this purpose. The fluorescence results establish that the phenomenon of binding of Ag(Hnor) complexes to HSA can be deduced from the static quenching mechanism. The results showed a significant binding propensity of the used Ag(I) compounds towards HSA. The role of the counteranion on the binding of Ag(I) compounds to HSA appeared to be remarkable. Compounds with (ClO_4_^−^) and (NO_3_^−^) were found to have the most efficient binding towards HSA as compared to BF_4_^−^and PF_6_^−^. Circular dichroism (CD) studies made clear that conformational changes in the secondary structure of HSA were induced by the presence of Ag(I) compounds. Also, the *α*-helical structure of HSA was found to get transformed into a *β*-sheeted structure. Interestingly, (ClO_4_^−^) and (NO_3_^−^) compounds were found to induce most substantial changes in the secondary structure of HSA. The outcome of this study may contribute to understanding the propensity of proteins involved in neurological diseases (such as Alzheimer's and Parkinson's diseases) to undergo a similar transition in the presence of Ag-*β*-carboline compounds.

## 1. Introduction

The history of silver applications started from its use in coins and jewelry. Women loved to decorate themselves with various trinkets of silver, but it is less well known that this metal can be an excellent metallotherapeutic agent. In the human body, silver (Ag) is not acting as an endogenous metal and exhibits relatively low toxicity. Ag(I) coordination compounds with a variety of ligands having nitrogen, phosphorus, and/or sulfur donor atoms have large applications in medicinal and analytical chemistry [1, 2]. Specifically, the antibacterial and antifungal actions of Ag(I) compounds are well known [3–7]. The properties of silver compounds, like aqueous solubility, light stability, and biological activity, can be modified by varying the number and types of organic ligands [1–7]. Furthermore, in the more recent past, Ag(I) compounds have also been reported to display antitumor activity and have shown activities comparable to the clinical chemotherapeutic drug (cisplatin) [8–11].

Human serum albumin (HSA) is responsible for about 60% of the plasma protein in humans and is accountable for nearly 80% of the osmotic pressure of the blood, and it plays a prominent role in drug disposition and efficacy [12]. Various drugs bind reversibly to albumin and other serum components, which thereby function as carriers [13]. Serum albumin is known to increase the solubility of many drugs in plasma and modulates their delivery to cells *in vivo* and *in vitro* [14–16]. Hence, it is relevant to study the interactions of drug candidates with this protein.

**Figure 1:**
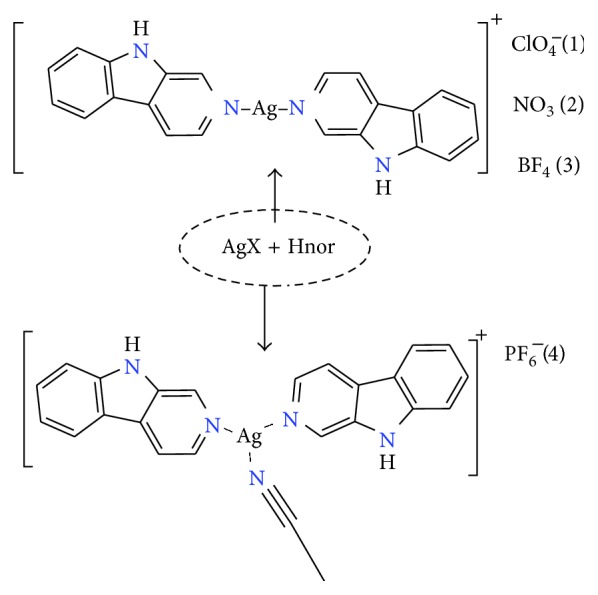
The structures of the ligand 9H-pyrido[3,4-b]indole (Hnor) and the four used Ag(I) compounds.

Norharmane, 9H-pyrido[3,4-b]indole, is a rather unconventional ligand, belonging to an alkaloid family called *β*-carbolines (*β*Cs). The molecule can act as a coordinating ligand, an H-bonding donor/acceptor, and may also act on *π*-*π* stacking; all these properties may act in synergy. A schematic chemical structure of norharmane is included in Figure 1.

*β*-Carbolines are present in many plants, arthropods, and insects. Endogenously, these alkaloids are synthesized by tryptophan or tryptophan-like indoleamines and also found in urine, plasma, platelets (∼0.1 nM), in the case of mammals. Interestingly, after alcohol intake and smoking, *β*-carboline concentrations are found to increase to ∼1.0 nM. It is also well known that some *β*-carboline derivates on photoexcitation induce chromosomal damages in mammal cells and may disarm viruses and bacteria [17]. Moreover, substituted aromatic *β*-carbolines may enter into the brain by crossing the blood-brain barrier (BBB) and may then be converted into methyl derivatives by specific enzymes, like methyltransferases. Some *β*-carbolines like 2,9-dimethyl-*β*-carbolines have exhibited mitochondrial damage leading to neurotoxicity, while 9-methyl-harmine has a neuroprotective effect. *β*-Carboline compounds are also known to reduce the expression of phosphorylated forms of the so-called tau protein, potently at multiple Alzheimer's disease-related sites [17–20].

In a previous study, some of us have shown that compounds of composition [Ag(Hnor)_2_](anion) display significant anticancer activity for the anions, namely, NO_3_^−^, ClO_4_^−^, BF_4_^−^, and PF_6_^−^, with the ClO_4_^−^ compound being even comparable to cisplatin in two different cancer cell lines [21]. To explore the possible molecular mechanism of action, we have decided to undertake a study of protein binding of the four silver compounds (**1–4**) with Hnor varying the ionic sphere by changing the counteranions, namely, using NO_3_^−^, ClO_4_^−^, BF_4_^−^, and PF_6_. HSA has been selected as a model for the protein binding studies. As spectroscopic techniques, UV–visible, fluorescence spectroscopy, and circular dichroism (CD) techniques have been chosen. Finally, the interactions of the Ag(I) compounds with the protein were studied using molecular modeling.

## 2. Experimental Section

### 2.1. Starting Materials and Syntheses

AgNO_3_, AgClO_4_, AgBF_4_, AgPF_6_, DL-tryptophan, and formaldehyde were purchased from Sigma-Aldrich. Human serum albumin (HSA; ≥99%, Sigma, USA) was essentially fatty-acid free and globulin free, purchased from Sigma, and used as received. We have synthesized the four Ag(I) compounds as described in before [21]. Other standard laboratory chemicals were used as available. Studies of protein folding have mainly been carried out in buffered dilute aqueous solutions to avoid loss of protein to the aggregation phenomenon. Stock solutions of HSA (300 *µ*M) and Ag(I) compounds were prepared in a mixture of dimethyl sulfoxide (5%) DMSO and 95% phosphate buffer (20 mM) of pH 7.4 (i.e., well above the isoelectric point of HSA, pH 4.7; hence, the protein possesses a net negative charge at this pH). Conductivity in solution was studied by using an Accumet AB30 Fisher Scientific conductometer at room temperature in MeOH solution of the compounds.

### 2.2. Protein Binding Studies

The samples of HSA were prepared in 20 mM phosphate buffer (pH 7.4), whereas silver complexes (1 mM) stock solution were prepared in DMSO and further diluted in 20 mM phosphate buffer to reach the desired concentration. In all the samples, the final concentration of DMSO was not more than 1%. The concentration of HSA was determined using the Beer–Lambert law with the molar extinction coefficient of 36500 M^−1^·cm^−1^ at 280 nm. To study structural changes in HSA by the addition of [Ag(I)(Hnor)_2_](anion) compounds, the UV absorption spectra were measured, by variation of the concentration of the [Ag(I)(Hnor)_2_](anion) compounds, while keeping the concentration of HSA constant. UV absorption spectra, from 240 nm to 320 nm, were recorded on a Perkin–Elmer Lambda 45 spectrophotometer at 25°C. Quartz cuvettes of 1 cm path length were used for the measurements. Fluorescence measurements were performed on a Hitachi spectrofluorometer (Model F 7000) equipped with a PC. The fluorescence spectra were collected at 25°C with a path length cell of 1 cm. The slit width used was 5 nm with a protein concentration of 5 *µ*M. The used excitation wavelength for the protein was 295 nm.

CD measurements were carried out on a Jasco spectropolarimeter (Model J-815) equipped with a microcomputer. The instrument was calibrated with D-10-camphorsulfonic acid. All the CD measurements were performed at 25°C with a thermostatically controlled cell holder, attached to a Neslab RTE-110 water bath with an accuracy of ±0.1°C. Spectra were collected with a scan speed of 0.2 nm/min and a response time of 1 s. Each spectrum was taken as the average of three scans. The far-UV CD spectra were measured at a protein concentration of 20 *µ*M at a path length of 1 cm.

All the spectra were recorded after equilibration of the reaction mixture for 5 min.

### 2.3. Molecular Docking

The rigid molecular docking studies were performed by using HEX 8.0.0 software [22], which is an interactive molecular graphics program for calculating and displaying possible docking modes of protein. The Hex 8.0.0 performs protein docking using spherical polar Fourier correlations [23]. Hex 8.0.0 necessitates the ligand and the receptor as input in PDB format. The parameters used for docking include the following: correlation type: shape only; FFT mode: 3D; grid dimension: 0.6; receptor range: 180; ligand range: 180; twist range: 360; and distance range: 40. The coordinates of compounds **1** and **2** were taken from its crystal structure as a .cif file and were converted to the PDB format using Mercury software. Te crystal structure of the human serum albumin (PDB ID: 1h9z) was downloaded from the protein data bank (http://www.rcsb.org./pdb). Visualization of minimum energy favorable docked poses has been performed using Discovery Studio 4.1 [24] and PyMOL [25].

## 3. Results and Discussion

### 3.1. General Observations and Synthesis

Synthesis of the four Ag(I) compounds, namely, [Ag(Hnor)_2_](ClO_4_) (**1**), [Ag(Hnor)_2_](NO_3_) (**2**), [Ag(Hnor)_2_(MeCN)](PF_6_) (**3**), and [Ag(Hnor)_2_](BF_4_) (**4**), were done by dissolving the starting Ag salt and Hnor in acetonitrile (ratio 1 : 2); they were characterized according to the procedures reported by some of us previously [21]. Conductivity studies in MeOH solution (1–5 mM) have shown that the compounds behave as 1 : 1 electrolytes. Given the known kinetic lability of Ag(I), it is assumed that the Hnor ligand may dissociate from and associate with the Ag ion, in solution, but on average, they are largely coordinated. The schematic structures of the ligand and the used Ag(I) compounds are depicted in Figure 1.

### 3.2. HSA Binding Studies

The interaction between the small bioactive molecules and protein receptors is a fundamental step in the drug discovery process. Obtaining a thorough idea of the interaction of the protein with chemical entities plays a vital role in the etiology of several diseases. The protein-drug intermediate products involved in governing various biochemical phenomena in both normal and diseased cells are known to play a significant role in metabolizing therapeutic compounds and their transport [26, 27].

#### 3.2.1. UV Absorption Studies

**Figure 2:**
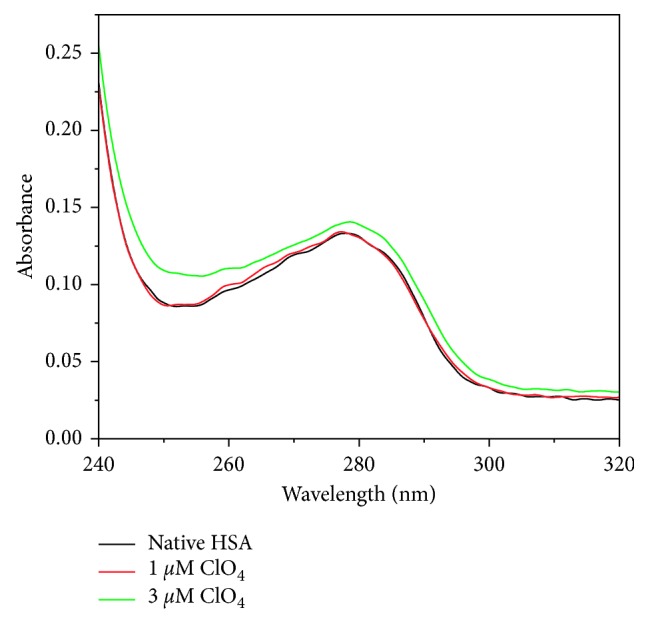
UV absorption difference spectra of HSA (5 *µ*M). These spectra represent the effect of compound 1 at 1 *µ*M and 3 *µ*M concentrations. (The difference spectrum was obtained by the HSA-Ag compound spectra minus Ag compound spectra).

The binding propensity of the drug candidate with the biomolecule was first studied by using the UV technique. The cumulative absorption of three aromatic amino acid residues gives rise to an absorption peak at 280 nm for human serum albumins (HSA) [28].  Figure 2 displays the UV absorption spectra of HSA in the absence and presence of [Ag(I)(Hnor)_2_]ClO_4_. The behavior of the other 3 compounds (compounds **2–4**) is quite similar, and these data are presented in Figure S1 (see Supplementary Materials).

With the concomitant increase in concentration of Ag(I) compounds, the absorbance of HSA increased, and shifts toward longer wavelengths were observed; the Ag(I) compounds give a definite pattern of the UV–Vis spectrum with weak absorbance at a higher concentration between 295 and 320 nm, ascribed to the ligand “Hnor.” The profound enhancement of UV absorbance (hyperchromism) with a redshift (bathochromic effect) of 7 nm (**1**), 5 nm (**2**), 5 nm (**3**), and 6 nm (**4**) in the spectra is suggestive of the formation of adducts/intermediates between the Ag(I) compounds and HSA. HSA is known to act as an important extracellular antioxidant, and this antioxidant property resides in one free cysteine-derived redox-active thiol (-SH) group (i.e., Cys34), which can occur in either reduced or oxidized form. Ag(I) being a soft Lewis acid, its compounds are known to have a high affinity towards sulfur-ligand atoms and moderate affinity towards nitrogen donor atoms. However, binding of Ag(I) towards methionine (Met)/histidine(His) residues and disulfide bridges, and nitrogen atoms, such as deprotonated peptide nitrogen, imine, and indole nitrogen, also cannot be ruled out completely [29, 30]. However, the compounds **1–4** are quite stable in the solution.

**Figure 3:**
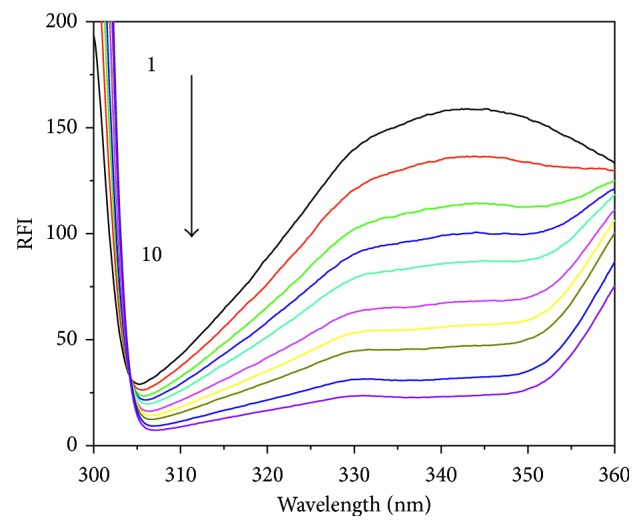
Fluorescence emission spectra of HSA (5 *µ*M) in the presence of various concentrations of [Ag(I)(Hnor)_2_]ClO_4_. Curves 1 to 10 correspond to compound concentrations of 0, 0.5, 1, 1.5, 2, 3, 4, 5, 7.5, and 10 *µ*M, respectively, when excited at 295 nm.

The difference spectra of HSA have confirmed that the conformational changes to HSA are due to binding of the Ag(I) compounds (Figure 3 and also Figure S2 in Supplementary Materials). Nevertheless, the variation in binding extent/mode exhibited by the spectra of the compounds to HSA may be associated with the effect of the counteranions. The anions may have facilitated the microenvironmental changes of protein and exposed the targeted site of a subdomain of protein to assist or enhance the selectivity of the metal center, that is, Ag(I). The effect of anions has been studied with the help of molecular docking to get further insight into their role. The possible role of anions has been discussed in computational studies section. However, the dissociation of the Ag(I) compound is a matter of main concern that we have also studied and found quite stable complex association of Ag(I)-Hnor. Shen et al. [31] have studied the interaction of Ag^+^ alone with HSA. When we compared these results of Ag compounds with Ag^+^ alone, silver compounds exhibited significantly worthy binding propensity compared to the Ag^+^ alone. In the next stage, with the aim to gain more information about the mode of binding of the Ag(I) compounds with HSA, solution fluorescence studies were carried out.

#### 3.2.2. Luminescence Studies

**Figure 4:**
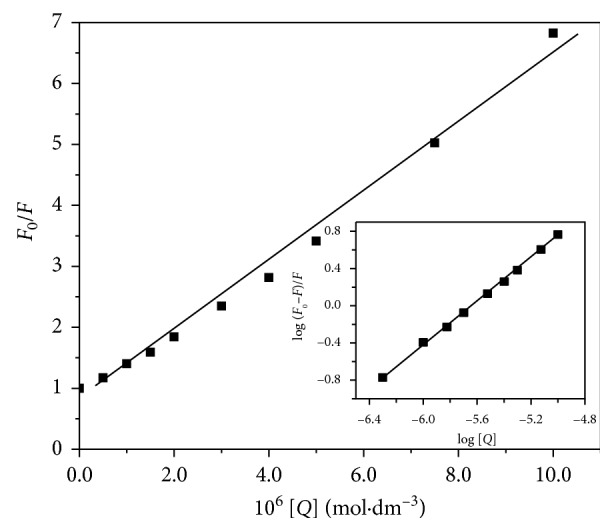
The Stern–Volmer plot of HSA fluorescence quenching by [Ag(I)(Hnor)_2_]ClO_4_ at 295 nm. Inset: plot of log (*F*_0_−*F*)/*F* as a function of log [complex].

The interaction between metal compounds and proteins has been widely investigated by using fluorescence spectroscopies [32–34]. The luminescence response of HSA, upon addition of different concentrations of Ag(I) compounds, was studied in 20 mM phosphate buffer by using an emission titration experiment. Preliminary luminescence studies of the four starting Ag compounds **1–4** have been reported earlier by some of us [21]. The excitation at 290 nm for all four compound results in a strong luminescence band at around 370 nm. The fluorescence studies of the ligand “Hnor” as well as the free silver salt, “AgNO_3_,” were also studied under the same conditions to compare the effect of separate entities with a combination of the two. Upon excitation at 295 nm, fluorescence intensity around 340 nm is known to reflect the changes of the tryptophan residue microenvironment [35]. Encouragingly, the luminescence of HSA was found substantially decreased in the presence of increasing concentrations of [Ag(I)(Hnor)_2_]ClO_4_, as depicted in Figure 4. In Figure S3, similar data are presented for compounds **2–4**, with only marginal differences upon anion variation.

A gradual decrease in the luminescence of HSA was observed upon increasing concentration of Ag(I) compounds showed significant redshift at the maximal emission wavelength of tyrosine(Tyr) and tryptophan(Trp) residues of 7 nm (**1**), 5 nm (**2**), 4 nm (**3**), and 4 nm (**4**), which indicates the altered conformation of the HSA by decrease in the polarity around Tyr and Trp residues and increase in hydrophobicity. The continuous, gradual addition of Ag(I) compounds resulted in further decreases in the fluorescence intensity of HSA, which again is indicative of a strong interaction between the Ag(I) compounds and HSA. The factors linked to quenching of the fluorescence can be associated with, for example, excited-state reaction, energy transfer, molecular rearrangements, collisional quenching, and/or ground-state compound formation [36, 37]. Therefore, the consequence of these molecular interactions enforcing towards the static quenching that comprises the establishment of a ground state adduct between the fluorophore and the quencher. The involvement of static quenching in the binding process can be determined with the help of analyzed values of bimolecular quenching constant (*K*_q_). A value of *K*_q_ higher than 2.0 × 10^10^ M^−1^·s^−1^ reflects the quenching to be static [38–40].

The interaction of the Ag compounds with HSA was quantified; the Stern–Volmer equation has been employed [41]:

(1) $$\begin{array}{c} \frac{F_{0}}{F}=1+K_{\text{SV}}\left[Q\right]=1+K_{\text{q}}\tau_{0}\left[Q\right], \end{array}$$

where *F*_0_ and *F* are the steady-state fluorescence intensities in the absence and presence of quencher at 340 nm, respectively. *K*_sv_ is the Stern–Volmer quenching constant, *K*_q_ stands for bimolecular quenching constant, *τ*_0_ is the lifetime of the fluorophore in the absence of quencher, and [*Q*] is the concentration of quencher (i.e., the Ag compound). By calculating the quenching rate constants, *K*_q_, one can distinguish between the static quenching and the dynamic quenching, and this was evaluated by using the following equation:

(2) $$\begin{array}{c} K_{\text{q}}=\frac{K_{\text{SV}}}{\tau_{0}}. \end{array}$$

**Figure 5:**
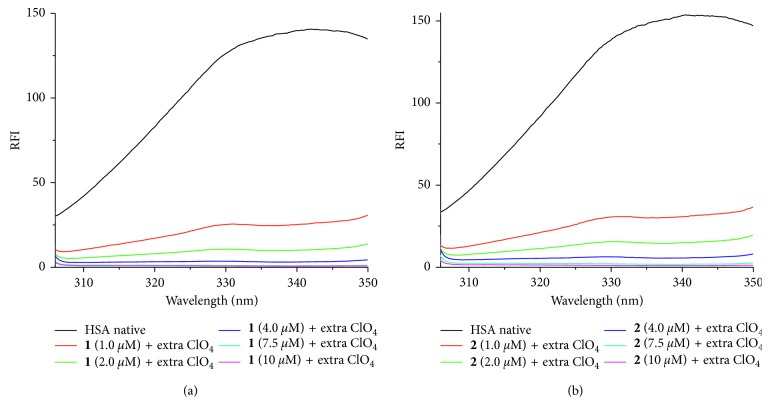
Fluorescence emission spectra of HSA (5 *µ*M) in the presence of various concentrations of (a) [Ag(I)(Hnor)_2_]ClO_4_ compound **1** + extra KClO_4_, and (b) [Ag(I)(Hnor)_2_]NO_3_ compound **2** + extra KClO_4_ corresponding to compound concentrations of 1.0, 2.0, 4.0, 7.5, and 10 *µ*M, respectively, when excited at 295 nm.

The value of tau(zero), *τ*_0_, for biopolymers is known to be 10^−8^ s. [33] The Stern–Volmer quenching constant (*K*_sv_) for the fluorometric titration of the Ag(I) compounds into HSA solution was calculated from the linear relationship between *F*_0_/*F* and [Ag(I)(Hnor)_2_]ClO_4_ (Figure 5) and enlisted in Table 1. In Figure S4, similar data are presented for compounds **2–4**, confirming the above observations.

Thus, these data ascertain the static quenching in the interaction of Ag(I) compounds with HSA, by the calculated value of *K*_q_. The equilibrium between free and bound molecules, when small molecules bind independently to a set of similar sites on a macromolecule, was ascertained by a modified Stern–Volmer equation [42]:

(3) $$\begin{array}{c} \log\frac{F_{0}-F}{F}=\log K+n\;\log\left[Q\right], \end{array}$$

where *K* and *n* are the binding constant and the number of binding sites, respectively. A plot of log(*F*_0_ − *F*/*F*) versus log[*Q*] was used to determine the value of *K* and *n* (inset in Figure 5). Several forces, like electrostatic, hydrogen bonds, weak van der Waals, hydrophobic, and steric contacts, can be thought of being responsible for the interaction between the albumin and Ag compounds. The value of the binding constant, *K*, was used to calculate the standard free energy change Δ*G*° of the binding of the ligand to the HSA, by using the relationship [42]:

(4) $$\begin{array}{c} \Delta G=-2.303\;\;\mathrm{RT}\;\;\log K. \end{array}$$

The values of *K*, *n*, and Δ*G* (binding) are presented in Table 1. All estimated values of *n* are approximately 1, indicating the existence of just one major binding site in HSA for the present Ag(I) compounds.

The extent of interaction of Ag(I) compounds with HSA was found in the order **1** (ClO_4_^−^) > **2** (NO_3_^−^) > **4** (BF_4_^−^) > **3** (PF_6_^−^), which can be associated with the differences in the effect of counteranions. Thus, compounds **1** (ClO_4_^−^) and **2** (NO_3_^−^) exhibit significantly higher binding affinities towards HSA, compared to other two compounds.

Since it is known that HSA is a monomeric, three-domain, allosteric protein with only one free cysteine, Cys34, the Ag(I) ion could selectively bind at this site because it has a strong preference for S-donor atoms. Also, it can be assumed that the counteranions have facilitated the microenvironmental changes and may have contributed to the exposure of Cys34 from the subdomain of HSA and facilitated that the sulfur atom of cysteine will coordinate to the Ag(I) center of the compounds. Nevertheless, the known affinity of Ag(I) for Met residues and disulfide bridges and nitrogen atoms of HSA coordination on such sites cannot be ignored completely. This hypothesis is supported by the observation that the extent of microenvironmental changes of the protein is much higher in the presence of additional ClO_4_^−^ and NO_3_^−^ anions (see below), and which agrees with the trend in their binding parameter (Table 1); thus, a more profound effect on the Ag(I) compound and its HSA interaction is observed.

### 3.3. Effects of Addition of Additional Anions

**Figure 6:**
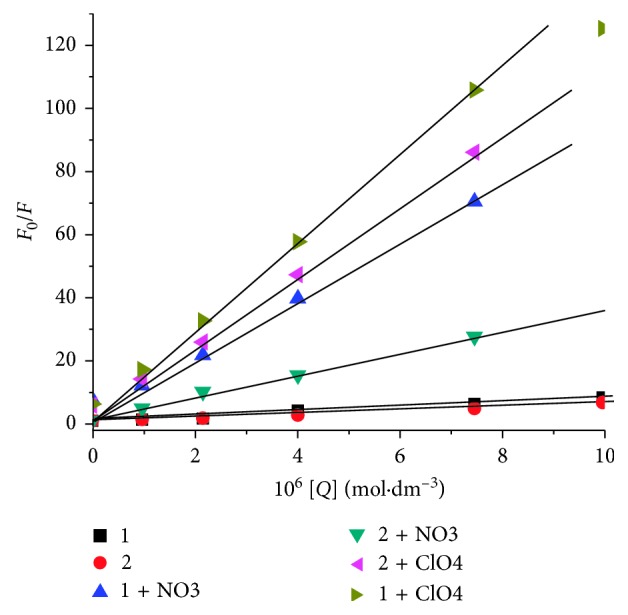
Stern–Volmer plot of HSA fluorescence quenching by (**1**) [Ag(I)(Hnor)_2_]ClO_4_ and (**2**) [Ag(I)(Hnor)_2_]NO_3_ with additional KClO_4_ and KNO_3_ at 295 nm.

**Figure 7:**
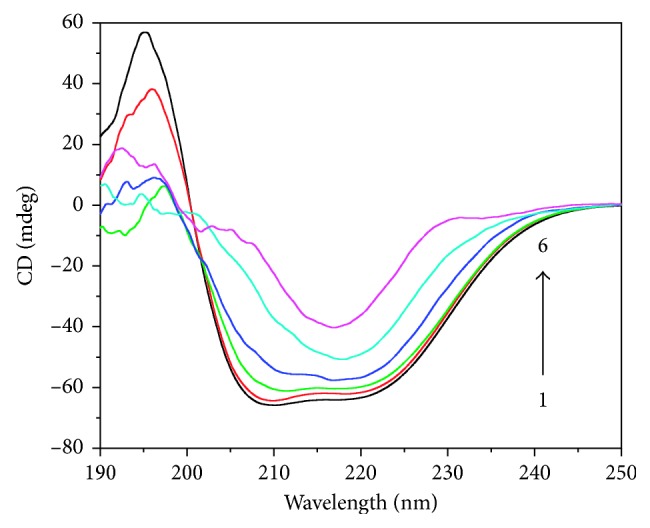
Far-UV CD spectra of HSA-compound system (HSA = 20 *µ*M) in the presence of various concentrations of [Ag(I)(Hnor)_2_]ClO_4_ complex. Curves from 1 to 6 correspond to compound concentrations of 0, 20, 40, 60, 80, and 100 mM, respectively.

Given the fact that the highest anticancer activity and also the most substantial HSA interaction takes place in case of the perchlorate and nitrate salts, it was decided to add extra perchlorate (and also nitrate) for all cases and to study the effect on the binding affinity. So, we carried out two sets of experiments and studied the binding propensity of Ag(I) compounds with HSA. In one experiment, the extra ClO_4_^−^ anion was added in a 1 : 4 ratio, compared to the Ag compound. In a second experiment, we used additional nitrate together with the Ag(I) compounds. Details are given in Figures 6 and 7, and the quite similar details for the other 3 compounds are presented in Figures S5 and S6 in Supplementary Materials.

To determine the role of only the anions (i.e., without Ag and Hnor), we also used KClO_4_ and KNO_3_ and studied their HSA binding by adding variable amounts of anions to the concentration maximum used in the experiments. We found that on the addition of KClO_4_ and KNO_3_ to HSA, only a negligible perturbance of the HSA structure has occurred. Hence, the effect of these anions (ClO_4_^−^ and KNO_3_^−^) on HSA conformation can be neglected in comparison to the effect of Ag compounds and Ag compounds + additional anions on HSA conformation. When the extra anions + compounds were titrated, the results obtained did show an exponential increase in binding affinity of the Ag(I) compounds.

These findings can be attributed to the enhanced exposure of the cysteine sulfur atoms of HSA, most likely caused by the significant electrostatic effect of the additional anions. This behavior is indicative of the increased microenvironmental changes of the HSA, thereby allowing the more binding of the Ag(I) center to coordinate to the HSA binding site.

The binding strength was evaluated by calculating the Stern–Volmer constant (*K*_sv_), a number of the binding sites (*n*) and Gibbs free energy (Δ*G*); (Table 2). It should be noted that the effect of ClO_4_^−^ is quite high, whereas NO_3_^−^ and BF_4_^−^ mutually exhibit a nearly similar level of effect, while the PF_6_^−^ anion comes at the end showing a smaller effect. However, no correlation with the size of these four anions is seen.

The effect of anions was also analyzed on the basis of binding constant (*K*) to study the binding affinity. The binding constant's value exhibited a vibrant increase in the presence of extra anions in particular with ClO_4_^−^ and NO_3_^−^ when compared with the results of complexes **1** and **2**. In complexes **1** and **2**, extra ClO_4_^−^ showed significant increase in the binding affinity as compared to extra NO_3_^−^ as evident from Table 2 (*K* values). This exponential increase of the magnitude of 10^4^ says a lot about the role of anions in the stronger binding affinity of the silver compounds with HSA. The order of binding of Ag(I) compounds with HSA upon extra anion addition was found to be **1** > **2** > **4** > **3**. Thus, it is evident that the number of the binding sites (*n*) increases upon addition of extra anions.

### 3.4. Binding and Conformational Changes in Circular Dichroism

**Figure 8:**
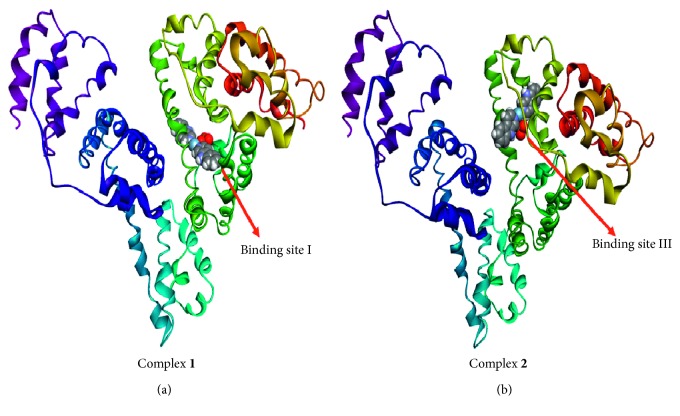
Binding of compounds (a) **1** and (b) **2** at different binding sites of HSA.

CD spectroscopy is an ideal technique for monitoring the conformational changes of proteins. As shown in Figure 8, the CD spectrum of free HSA (line 1) exhibits two negative bands at 208 nm and 222 nm in the ultraviolet region, attributed to *n*-*π* ^∗^ transfer for the peptide bond, with a positive ellipticity at 193 nm, which is the characteristic of an *α*-helix structure of HSA [43]. CD often allows obtaining information for almost all secondary structural variants, such as *α*-helices, *β*-sheets, *β*-turns, and random coil structures. All of these structures provide origins to bands of distinct forms and degrees in the far-UV region. Modifications of the ellipticity at 222 nm (−MRE222) are convenient methods for observing and quantifying changes in the *α*-helical content [44]. CD spectra recorded in the presence and absence of various concentrations of Ag(I) compounds are presented in Figure 8. Similar CD curves for the other three compounds are shown in Figure S8, as they show comparable behavior.

The CD signal, expressed in millidegree, obtained over the wavelength range of 190–250 nm, was converted to a mean residue ellipticity (MRE, θ), using the following conversion:

(5) $$\begin{array}{c} \text{MRE}=\frac{\theta_{\text{obs}}\;\left(\text{mdeg}\right)}{\left(10\times n\times C_{p}\times l\right)}, \end{array}$$

where *θ*_obs_ is the CD in millidegree, the number of amino acid residues (585) is given by *n*, “*l*” is the path length (cm), and *C*_*p*_ is the molarity. The unit of MRE is deg·cm^2^·dmol^−1^.

The *α*-helix contents of free and combined HSA were calculated from the MRE value at 222 nm, using the following equation [43, 45]:

(6) $$\begin{array}{c} \%\;\alpha -\text{helix}=\left[\frac{{\text{MRE}}_{222\text{ nm}}-2,340}{30,300}\right]\times 100. \end{array}$$

With increasing concentrations of the Ag(I) compounds, the CD signal exhibits significant changes in ellipticity, and this change corroborates with the binding of the compounds with the HSA backbone (spectra 2–6, Figure 8). In fact with increasing concentration of the compounds the ellipticity decreases. However, at high concentration of silver compounds, a transition of the secondary structure (i.e., *α*-helix to *β*-sheet) was observed.

The results confirm that the compound binds to the amino acid residues of the primary polypeptide chain in HSA, which has distorted its hydrogen-bonding networks. Interestingly, the decrease in the *α*-helical content is indicative of substantial microenvironmental changes of the polypeptide chains of HSA, which increased the exposure of some hydrophobic regions that were previously submerged. The microenvironmental changes of the protein were found more pronounced in the case of **1** (ClO_4_^−^) and **2** (NO_3_^−^), as compared to other two compounds (Table 3). The results of all studies corroborate well with the findings of fluorescence and UV studies.

### 3.5. Molecular Docking

**Figure 9:**
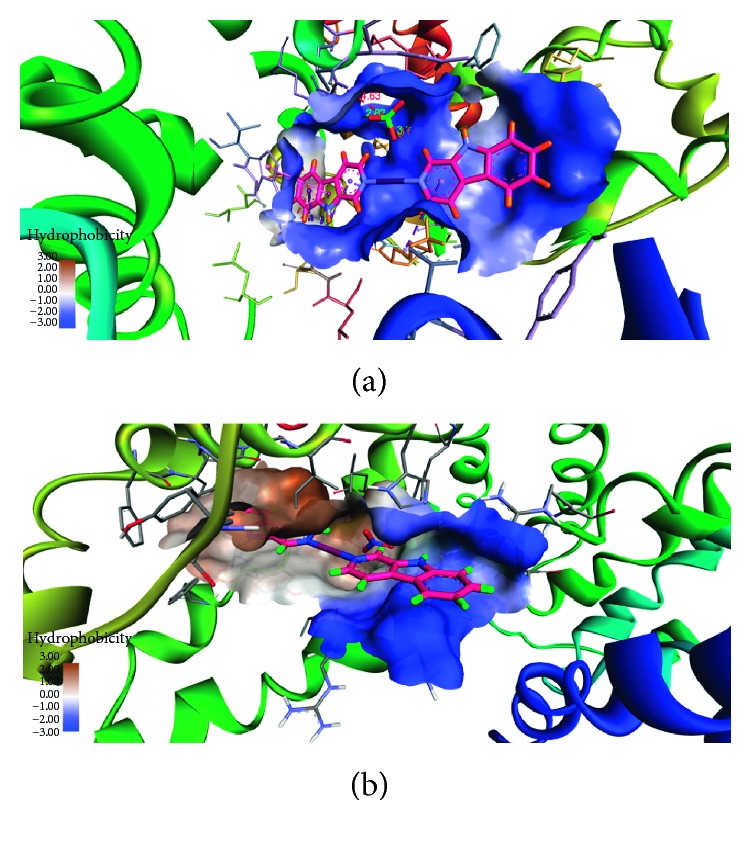
(a) Molecular docked model of compound **1** (stick representation) located within the hydrophobic pocket in the subdomain IIA of HSA; (b) molecular docked model of compound **2** (stick representation) located within the hydrophobic pocket in the subdomain IB of HSA.

**Figure 10:**
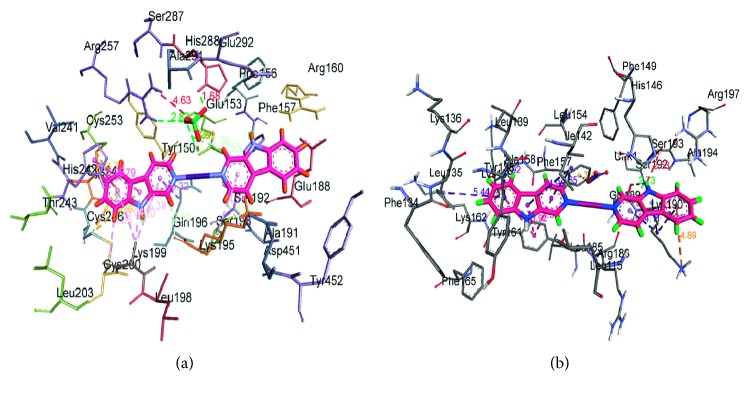
Noncovalent interactions of compounds (a) **1** and (b) **2** at the subdomains IIA and IB of HSA, respectively.

To provide further and deeper insight into the interactions of HSA with compounds **1** and **2**, a molecular docking technique was employed to learn more about the exact binding sites inside the molecular target HSA. From the 3D structure of crystalline albumin, it is known that HSA comprises three homologous domains that assemble to form a heart-shaped molecule, denoted I, II, and III: I (residues 1–195), II (196–383), and III (384–585). The principal region of compound **1** binding sites of HSA is located in hydrophobic cavities in the subdomains IIA and IIIA, corresponding to sites I and II, respectively, and the tryptophan residue (Trp-214) of HSA in the subdomain IIA. A large hydrophobic cavity in the subdomain IIA (a binding site at I) to accommodate compound **1** is present, while compound **2** preferentially appears to the bind at site III. The minimum energy docked pattern (Figure 9(a)) indicates that compound **1** is primarily located within the subdomain IIA of HSA, forming numerous hydrophobic contacts (*π*-σ, *π*-*π* stacked, and *π*-alkyl) with GLN196, HIS242, LYS199, LYS195, CYS200, CYS245, and CYS245 residues of the hydrophobic binding site IIA (Figure 10(a)). Furthermore, also a number of hydrogen bonds and specific electrostatic interaction formed by the compound **1** are observed (Table 4).

The resulting docked pattern (Figure 9(b)) indicates that compound **2** is located in the subdomains IA and IB, forming various noncovalent interactions like hydrogen bond, electrostatic, and hydrophobic within the binding cavity residues. A detailed description is given in Table 5 (Figure 10(b)). These noncovalent interactions formed by both the compounds **1** and **2** are dominated by hydrophobic contacts, with additional stabilization also assisted by the hydrogen bonding and electrostatic interaction with the polar residues of the binding site cavity. It has been previously observed [46, 47] that hydrogen bonding and electrostatic interaction decreased the hydrophilicity and increased the hydrophobicity to keep the compounds-HSA system stable. The relative binding energy of the docked structures was found to be −344.74 and −326.31 kJ/mol for **1** and **2**, respectively. The results obtained from the molecular docking studies revealed that the hydrophobic forces dominated the interaction of Ag compounds with the HSA. Notably, the binding of the compounds **1** and **2** to different sites can be largely associated with the difference of ionic sphere.

To find out the effect of counterion in the HSA binding, we also perform the docking of the individual counterion (nitrate ion and perchlorate ion) with the HSA (Figure S9). In the minimum energy docked pose, the nitrate and perchlorate ion are found in the outer environment of the binding site's IIA and IIIA domains, respectively. These anions form electrostatic and other noncovalent interaction at these domains, which could be responsible for the microenvironmental changes or conformational changes of proteins. So that presence of the nitrate and perchlorate ions in the complex may have facilitated or enhanced the binding propensity (see Tables S1 and S2 in Supplementary Materials), which is also evident from the binding constant values in the presence of the anions. Thus, it plays an influential role in binding to the biomolecule. This observation confirms the significant role of the anions in the mode of interaction with the biomolecule.

## 4. Concluding Remarks

*β*-Carbolines motif crosses blood-brain membrane (BBM) and are known to inhibit the phosphorylated form of the tau protein [9]. These inhibitions are the essential features to study for any new compound to treat diseases such as Alzheimer's disease. Spectrophotometric methods have been used to investigate the interaction between HSA and the present Ag(I) compounds. All Ag(I) compounds showed a strong affinity towards HSA. An extra effect of the perchlorate and nitrate anions was found in the binding studies, which ascertained the higher binding propensity of the Ag(I) compounds with counteranions ClO_4_^−^ and NO_3_^−^. The moderate HSA binding in the case of BF_4_^−^ and PF_6_^−^ salts could be enhanced by the addition of extra anions, namely, ClO_4_^−^ and NO_3_^−^. The results have shown a significant role of the anion in Ag-HSA binding and even showed an exponential increase in the binding propensity of the Ag(I) compounds. This affinity has been attributed to the increased exposure of the Ag-binding subdomains of HSA, as a result of the electrostatic and hydrophobic interactions. The presence of Hnor, despite being a labile ligand, appears to be important for the binding to HSA. The effect of anions was further validated by the molecular modeling studies which corroborate well with our findings of binding studies and ascertain that anions play an importance role in binding to HSA.

## Figures and Tables

**Table 1:** Stern–Volmer quenching constants and bimolecular quenching rate constant for the interaction of HSA with four Ag(I) Hnor compounds.

Complex	*K*_SV_ (10^5^) (M^−1^)	*K*_q_ (10^12^) (M^−1^·s^−1^)	*K* (10^6^) (M^−1^)	*n*	∆*G* (kJ mol^−1^)
**1** (ClO_4_^−^)	5.37	53.7	3.74	1.1	−37.50
**2** (NO_3_^−^)	5.34	53.4	3.64	1.0	−37.44
**3** (PF_6_^−^)	4.53	45.3	0.55	1.2	−32.73
**4** (BF_4_^−^)	3.97	39.7	1.14	1.1	−34.55
Hnor	2.95	29.5	0.33	1.0	−31.40
AgNO_3_	3.10	31.0	1.94	1.1	−35.88

**Table 2:** Parameters for the interaction of HSA with Ag(I) compounds with the addition of extra anion, namely, ClO_4_^−^ and NO_3_^−^.

Complex	*K*_SV_ (10^7^) (M^−1^)	*K* (10^10^) (M^−1^)	*n*	∆*G* (kJ mol^−1^)
*Set A*				
**1** (+extra ClO_4_^−^)	2.21	17.2	1.77	−64.12
**2** (+extra ClO_4_^−^)	1.29	6.05	1.58	−57.48
**3** (+extra ClO_4_^−^)	0.84	0.46	1.55	−55.13
**4** (+extra ClO_4_^−^)	1.85	1.19	1.69	−61.52
*Set B*				
**1** (+extra NO_3_^−^)	2.06	3.64	1.64	−60.26
**2** (+extra NO_3_^−^)	1.24	0.66	1.53	−56.05
**3** (+extra NO_3_^−^)	0.80	0.08	1.41	−51.01
**4** (+extra NO_3_^−^)	1.38	0.38	1.48	−54.67

**Table 3:** Effect of the Ag(I) compounds on the *α*-helical structure of HSA (model protein) expressed as percentage *α*-helix character.

Concentration (mM)	% *α*-helix			
	**1** (ClO_4_^−^)	**2** (NO_3_^−^)	**3** (PF_6_^−^)	**4** (BF_4_^−^)
0	67.03	67.03	67.03	67.03
20	64.07	62.85	63.57	65.25
40	62.23	61.61	62.57	63.82
60	58.59	58.41	60.37	61.53
80	50.18	52.02	56.31	56.66
100	35.63	42.73	47.18	47.52

**Table 4:** Noncovalent interactions of compound **1** with the HSA binding site IIA.

Name	Distance (Å)	Category	Type
ARG257: H11-Complex **1**: O1′	2.81	Hydrogen bond	Conventional
HIS288: H1-compound **1**: O3	1.68		
Compound **1**: O1′-GLU153: O1	3.24		
Compound **1**: Cl1-GLU153: O1	3.63	Electrostatic	Attractive charge
GLN196-compound **1**	3.32	Hydrophobic	*π*-Sigma
HIS242-compound **1**	4.78		*π*-*π* Stacked
HIS242-compound **1**	3.92		*π*-*π* Stacked
Compound **1**-LYS199	5.38		*π*-Alkyl
Compound **1**-LYS199	4.19		*π*-Alkyl
Compound **1**-LYS195	4.75		*π*-Alkyl
Compound **1**-LYS199	5.03		*π*-Alkyl
Compound **1**-CYS200	5.25		*π*-Alkyl
Compound **1**-CYS245	4.23		*π*-Alkyl
Compound **1**-CYS246	4.92		*π*-Alkyl

**Table 5:** Noncovalent interactions of compound **2** with the HSA binding site III.

Name	Distance (Å)	Category	Type
Compound **2**: H4-GLY189:O	2.73	Hydrogen bond	Conventional
LYS190-compound **2**	4.88	Electrostatic	*π*-Cation
Compound **2**: O3-PHE157	3.39	Electrostatic	*π*-Anion
TYR161-compound **2**	4.29	Hydrophobic	*π*-*π* Stacked
TYR161-compound **2**	4.72		*π*-*π* Stacked
Compound **2**-A: TYR161	3.91		*π*-*π* Stacked
Compound **2**-A: ILE142	3.64		*π*-Alkyl
Compound **2**-A: ILE142	4.98		*π*-Alkyl
Compound **2**-A: LEU135	5.43		*π*-Alkyl
Compound **2**-A: ALA158	4.91		*π*-Alkyl
Compound **2**-A: LYS190	4.11		*π*-Alkyl
Compound **2**-A: LYS190	3.09		*π*-Alkyl
Compound **2**-A: LYS190	3.21		*π*-Alkyl

## Abbreviations

HSA: Human serum albumin
CD: Circular dichroism
Cys: Cysteine
Met: Methionine
Hnor: 9H-pyrido[3,4-b]indole (norharmane).

## Supplementary Materials

Supplementary Materials Figure S1: UV absorption spectra of HSA in the absence and presence of compounds **1–4**; concentration [HSA] = 5 *μ*M. Figure S2: UV absorption difference of HSA (the difference UV absorption spectra were obtained by HSA-Ag compound spectra minus Ag compound spectra). Figure S3: fluorescence emission spectra of HSA (5 *μ*M) in the presence of various concentrations of compounds **2**, **3**, and **4**; curves from 1 to 10 correspond to compound concentrations of 0, 0.5, 1, 1.5, 2, 3, 4, 5, 7.5, and 10 *µ*M, respectively, when excited at 295 nm. Figure S4: the Stern–Volmer plot for the quenching of the HSA fluorescence by compounds **2**, **3**, and **4** at 295 nm. *Inset*: plot of log (*F*_0_–*F*)/*F* as a function of log (complex). Figure S5: fluorescence emission spectra of HSA (5 *μ*M) in the presence of various concentrations of compounds **1–4**, corresponding to compound concentrations of 0, 1, 2, 4, 7.5, and 10 *µ*M with the addition of extra KClO_4_ in a 1 : 4 ratio, respectively, when excited at 295 nm. Figure S6: fluorescence emission spectra of HSA (5 *μ*M) in the presence of various concentrations of compounds **1–4**, corresponding to compound concentrations of 0, 1, 2, 4, 7.5, and 10 *µ*M with the addition of extra KNO_3_ in 1 : 4 ratio, respectively, when excited at 295 nm. Figure S7: CD spectra of the HSA-compound system (HSA = 20 *μ*M) in the presence of various concentrations of compounds **2**, **3**, and **4**; curves from 1 to 6 corresponding to compound concentrations of 0, 20, 40, 60, 80, and 100 mM, respectively. Figure S8: absorption spectra of Ag complexes only, 1: ClO_4_ (red line) and 2: NO_3_ (black line) of 1.0 mM solution. Figure S9: molecular docked model of HSA in presence of anions. Table S1: noncovalent interactions of nitrate ion with the HSA. Table S2: noncovalent interactions of perchlorate ion with the HSA.
