# Supplementary material for: β-Carboline Silver Compound Binding Studies with Human Serum Albumin: A Comprehensive Multispectroscopic Analysis and Molecular Modeling Study
Source: Bioinorg Chem Appl. 2018 Mar 25;2018:9782419. doi: 10.1155/2018/9782419 (PMC5889910; doi:10.1155/2018/9782419)
Supplement: Supplementary Materials — Figure S1: UV absorption spectra of HSA in the absence and presence of compounds 1–4; concentration [HSA] = 5 μM. Figure S2: UV absorption difference of HSA (the difference UV absorption spectra were obtained by HSA-Ag compound spectra minus Ag compound spectra). Figure S3: fluorescence emission spectra of HSA (5 μM) in the presence of various concentrations of compounds 2, 3, and 4; curves from 1 to 10 correspond to compound concentrations of 0, 0.5, 1, 1.5, 2, 3, 4, 5, 7.5, and 10 µM, respectively, when excited at 295 nm. Figure S4: the Stern–Volmer plot for the quenching of the HSA fluorescence by compounds 2, 3, and 4 at 295 nm. Inset: plot of log (F 0–F)/F as a function of log (complex). Figure S5: fluorescence emission spectra of HSA (5 μM) in the presence of various concentrations of compounds 1–4, corresponding to compound concentrations of 0, 1, 2, 4, 7.5, and 10 µM with the addition of extra KClO4 in a 1 : 4 ratio, respectively, when excited at 295 nm. Figure S6: fluorescence emission spectra of HSA (5 μM) in the presence of various concentrations of compounds 1–4, corresponding to compound concentrations of 0, 1, 2, 4, 7.5, and 10 µM with the addition of extra KNO3 in 1 : 4 ratio, respectively, when excited at 295 nm. Figure S7: CD spectra of the HSA-compound system (HSA = 20 μM) in the presence of various concentrations of compounds 2, 3, and 4; curves from 1 to 6 corresponding to compound concentrations of 0, 20, 40, 60, 80, and 100 mM, respectively. Figure S8: absorption spectra of Ag complexes only, 1: ClO4 (red line) and 2: NO3 (black line) of 1.0 mM solution. Figure S9: molecular docked model of HSA in presence of anions. Table S1: noncovalent interactions of nitrate ion with the HSA. Table S2: noncovalent interactions of perchlorate ion with the HSA. [file 9782419.f1.doc]

***Supporting Information to the Paper:***

**β-Carboline Silver Compounds Binding Studies with Human Serum Albumin: A Comprehensive Multi-spectroscopic Analysis and Molecular Modelling Study**

Ali Alsalme,^a†*^ Rais Ahmad Khan,^a†*^ Arwa M. Alkathiri,^a^ Mohd. Sajid Ali,^b^ Sartaj Tabassum, ^b^ Mohammed Jaafar,^a^ and Hamad Al-lohedan,^b^

^a^*Department of Chemistry, College of Science, King Saud University, P.O. Box 2455 Riyadh 11451, Kingdom of Saudi Arabia.*

*^b^Surfactant Research Chair, Department of Chemistry, College of Science, King Saud University, P.O. Box 2455 Riyadh 11451, Kingdom of Saudi Arabia.*

**Corresponding Author: Dr. Ali Alsalme, email:* [*aalsalme@ksu.edu.sa*](mailto:aalsalme@ksu.edu.sa) *Phone: +966540518430* and *Dr. Rais Ahmad Khan, email:* [*raischem@gmail.com*](../../../../../Downloads/raischem@gmail.com)

*†Both authors contributed equally.*

**Table of Contents**

**Figure S1:** UV absorption spectra of HSA in the absence and presence of compounds **1-4**; concentration [HSA] = 5 μM.

**Figure S2:** UV absorption difference of HSA (The difference UV absorption spectra was obtained by HSA-Ag compound spectra minus Ag compound spectra).

**Figure S3.** Fluorescence emission spectra of HSA (5 μM) in the presence of various concentrations of compounds **2, 3** and **4**, curves from 1 to 10 correspond to compound concentrations of 0, 0.5. 1, 1.5, 2, 3, 4, 5, 7.5 and 10 µM, respectively, when excited at 295 nm.

**Figure S4.** The Stern-Volmer plot for the quenching of the HSA fluorescence by compounds **2**, **3** and **4** at 295 nm*. Inset*: Plot of log (F_0_ - F)/F as a function of log [complex]***.***

**Figure S5**. Fluorescence emission spectra of HSA (5 μM) in the presence of various concentrations of compoundsd **1**- **4**, corresponding to compound concentrations of 0, 1, 2, 4, 7.5 and 10 µM with the addition of extra KClO_4_ in a 1:4 ratio, respectively, when excited at 295 nm.

**Figure S6.** Fluorescence emission spectra of HSA (5 μM) in the presence of various concentrations of compounds **1**- **4**, corresponding to compound concentrations of 0, 1, 2, 4, 7.5 and 10 µM with the addition of extra KNO_3_ in 1:4 ratio, respectively, when excited at 295 nm**.**

**Figure S7.** CD spectra of the HSA–compound system (HSA = 20 μM) in the presence of various concentrations of compounds **2**, **3** and **4**; curves from 1 to 6 corresponding to compound concentrations of 0, 20, 40, 60, 80, and 100 mM, respectively.

**Figure S8**. Absorption spectra of Ag complexes only, **1:** ClO_4_ (red line) and **2:** NO_3_ (black line)

**Table S1.** Non-covalent interactions of nitrate ion with the HSA.

| **Name** | **Distance (A^o^)** | **Category** | **Type** |
| --- | --- | --- | --- |
| A:ARG218:NH2 - :nitrate:O  :nitrate:N - A:GLU450:OE1  A:VAL343:HN - :nitrate:O  A:SER342:CA - :nitrate:O | 4.31442  3.67189  2.4717  3.05071 | Electrostatic  Electrostatic  Hydrogen Bond  Hydrogen Bond | Attractive Charge  Attractive Charge  Conventional Hydrogen Bond  Carbon Hydrogen Bond |

**Table S2.** Non-covalent interactions of perchlorate ion with the HSA.

| **Name** | **Distance (A^o^)** | **Category** | **Type** |
| --- | --- | --- | --- |
| A:HIS464:HD1 - :perchlorate:O  : perchlorate:O - A:CYS461:O  : perchlorate:O - A:THR474:O  A:THR474:CA - : perchlorate:O | 1.98494  2.99047  2.96572  3.58332 | Hydrogen Bond  Hydrogen Bond  Hydrogen Bond  Hydrogen Bond | Conventional Hydrogen Bond  Conventional Hydrogen Bond  Conventional Hydrogen Bond  Carbon Hydrogen Bond |

**Figure S1.** UV absorption spectra of HSA in the absence and presence of compounds **1-4**; concentration [HSA] = 5 μM.

**Figure S2:** Difference (HSA-Ag compound spectra minus Ag compound spectra) UV absorption spectra for all **2**-**4** compounds and native HSA.

**Figure S3.** Fluorescence emission spectra of HSA (5 μM) in the presence of various concentrations of compounds **2, 3** and **4**, curves from 1 to 10 correspond to compound concentrations of 0, 0.5. 1, 1.5, 2, 3, 4, 5, 7.5 and 10 µM, respectively, when excited at 295 nm.

**Figure S4.** The Stern-Volmer plot for the quenching of the HSA fluorescence by compounds **2**, **3** and **4** at 295 nm*. Inset*: Plot of log (F_0_ - F)/F as a function of log [complex]***.***

**Figure S5**. Fluorescence emission spectra of HSA (5 μM) in the presence of various concentrations of compounds **1**- **4**, corresponding to compound concentrations of 0, 1, 2, 4, 7.5 and 10 µM with the addition of extra KClO_4_ in a 1:4 ratio, respectively, when excited at 295 nm.

**Figure S6.** Fluorescence emission spectra of HSA (5 μM) in the presence of various concentrations of compounds **1**- **4**, corresponding to compound concentrations of 0, 1, 2, 4, 7.5 and 10 µM with the addition of extra KNO_3_ in 1:4 ratio, respectively, when excited at 295 nm**.**

**Figure S7.** CD spectra of HSA–compound system (HSA = 0.8 mg/ml) in presence of various concentrations of compounds **2**, **3** and **4**; curves from 1 to 6 corresponding to compound concentrations of 0, 20, 40, 60, 80, and 100 mM, respectively.

**Figure S8**. Absorption spectra of Ag complexes only, **1:** ClO_4_ (red line) and **2:** NO_3_ (black line) of 1.0 mM solution.


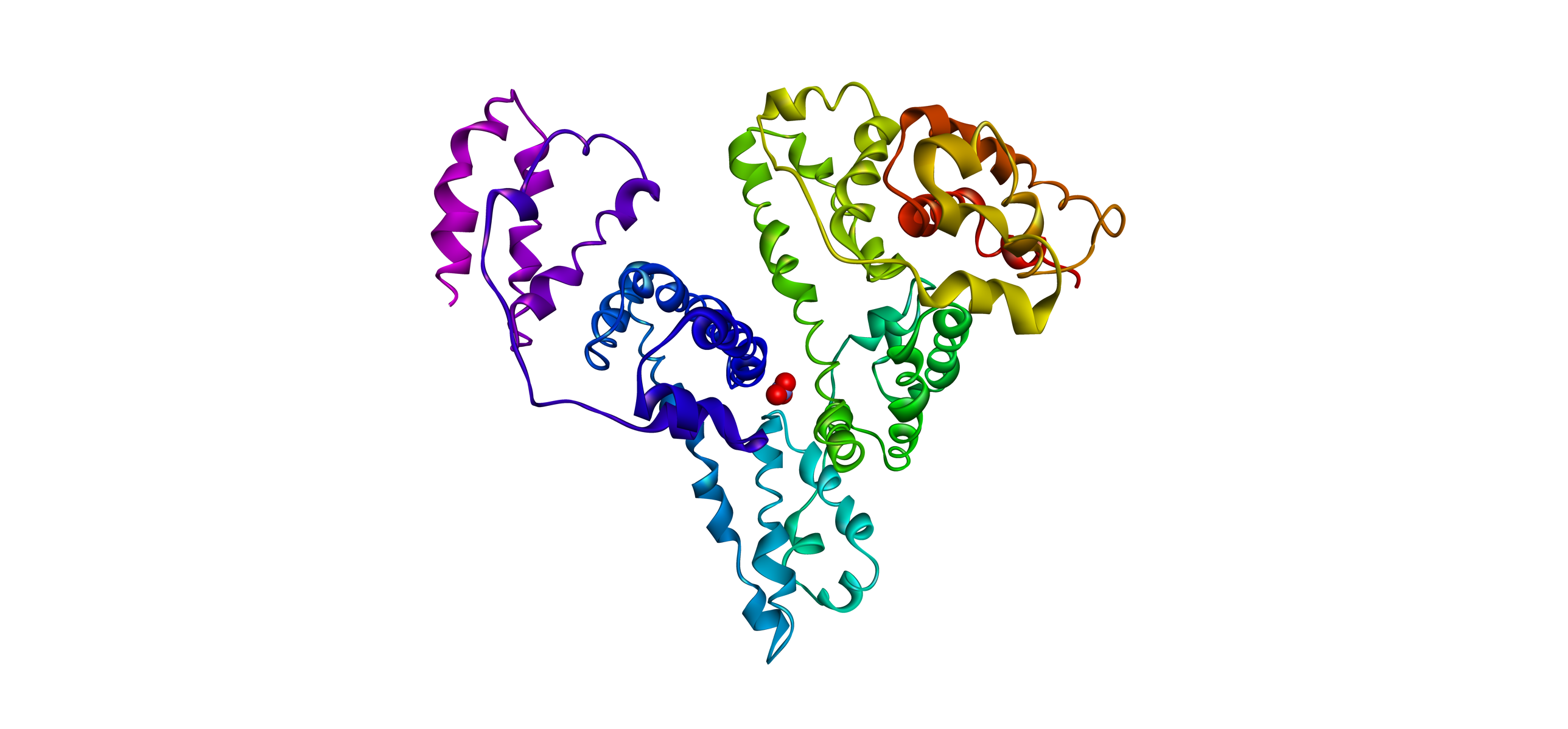

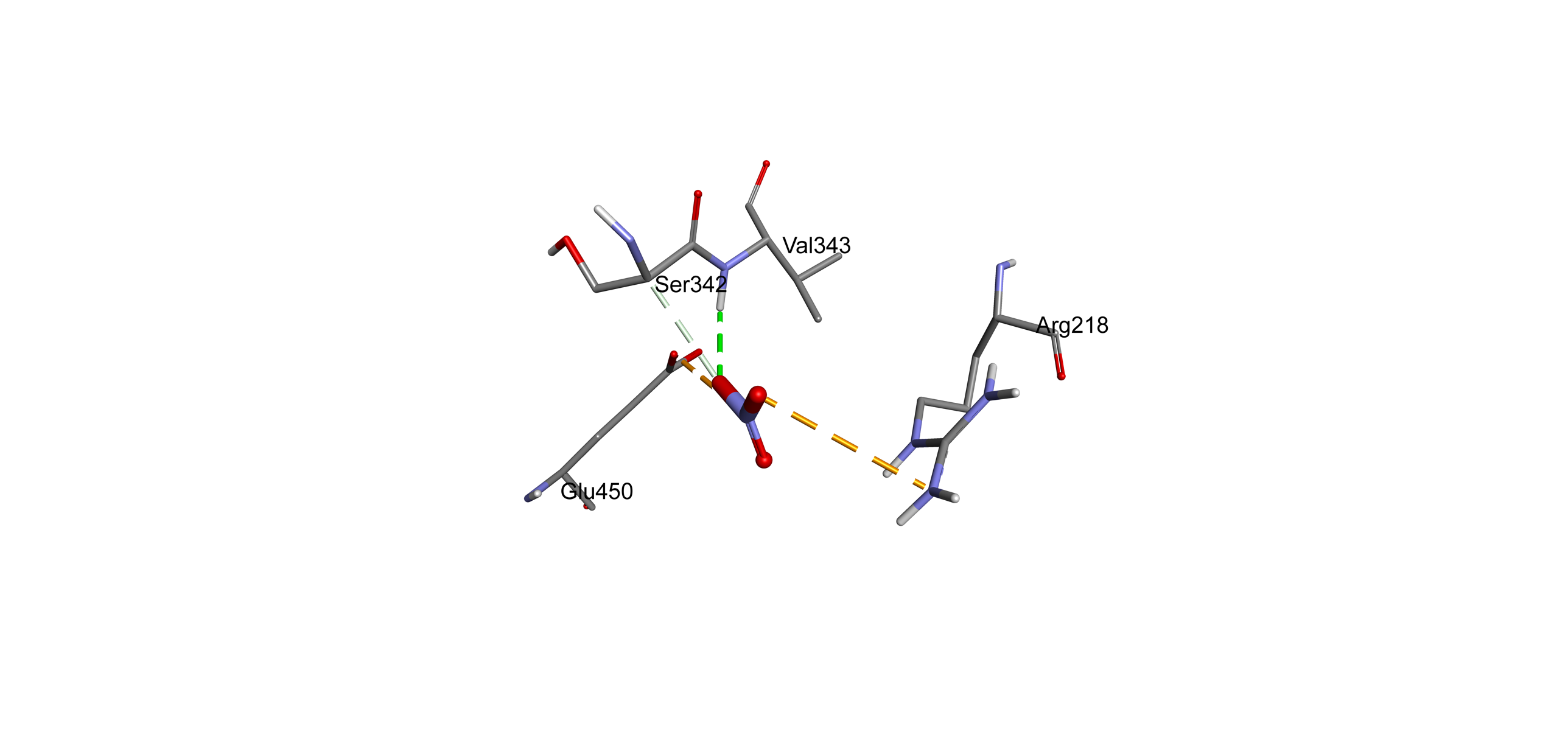


(A) Molecular docked model of HSA + NO_3_^-^


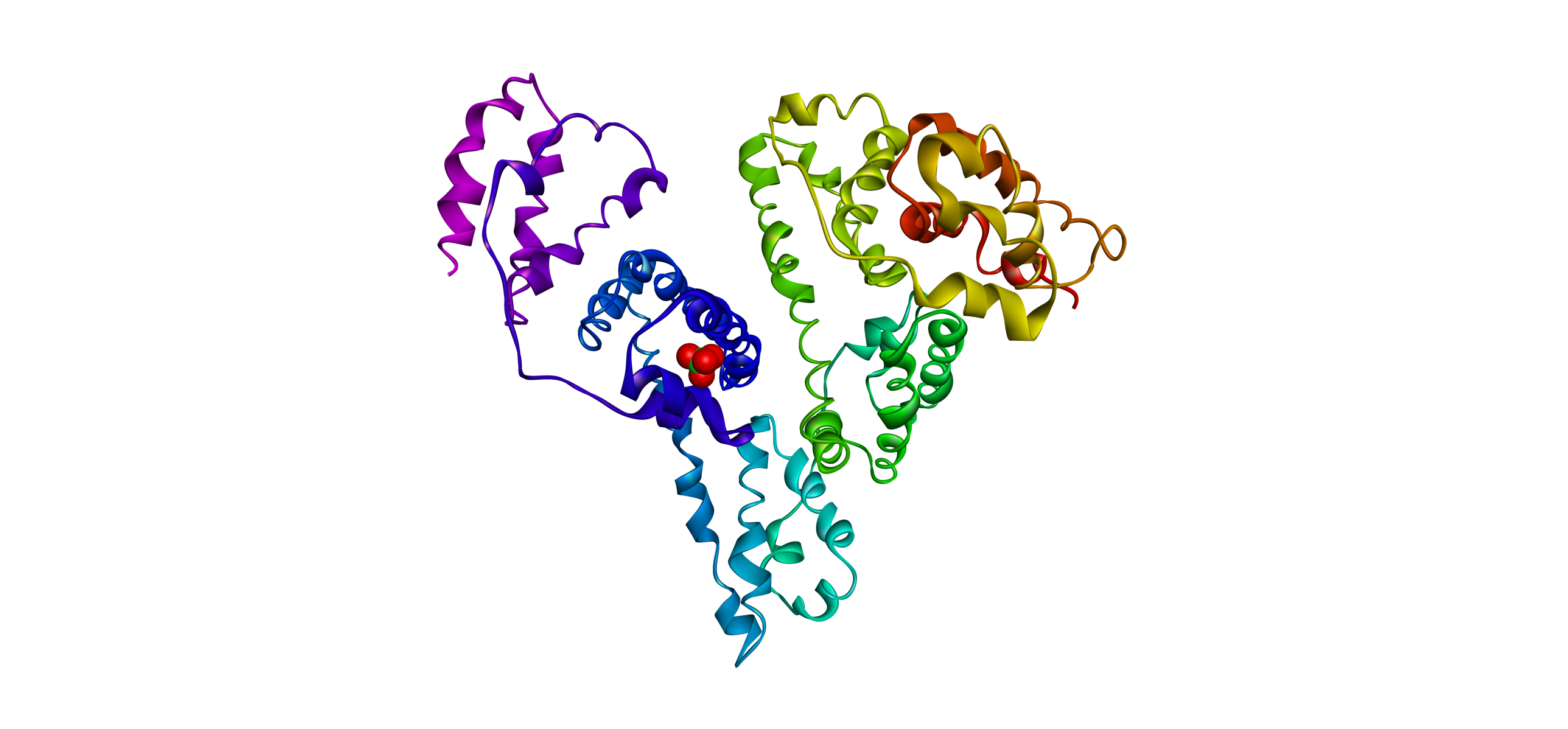

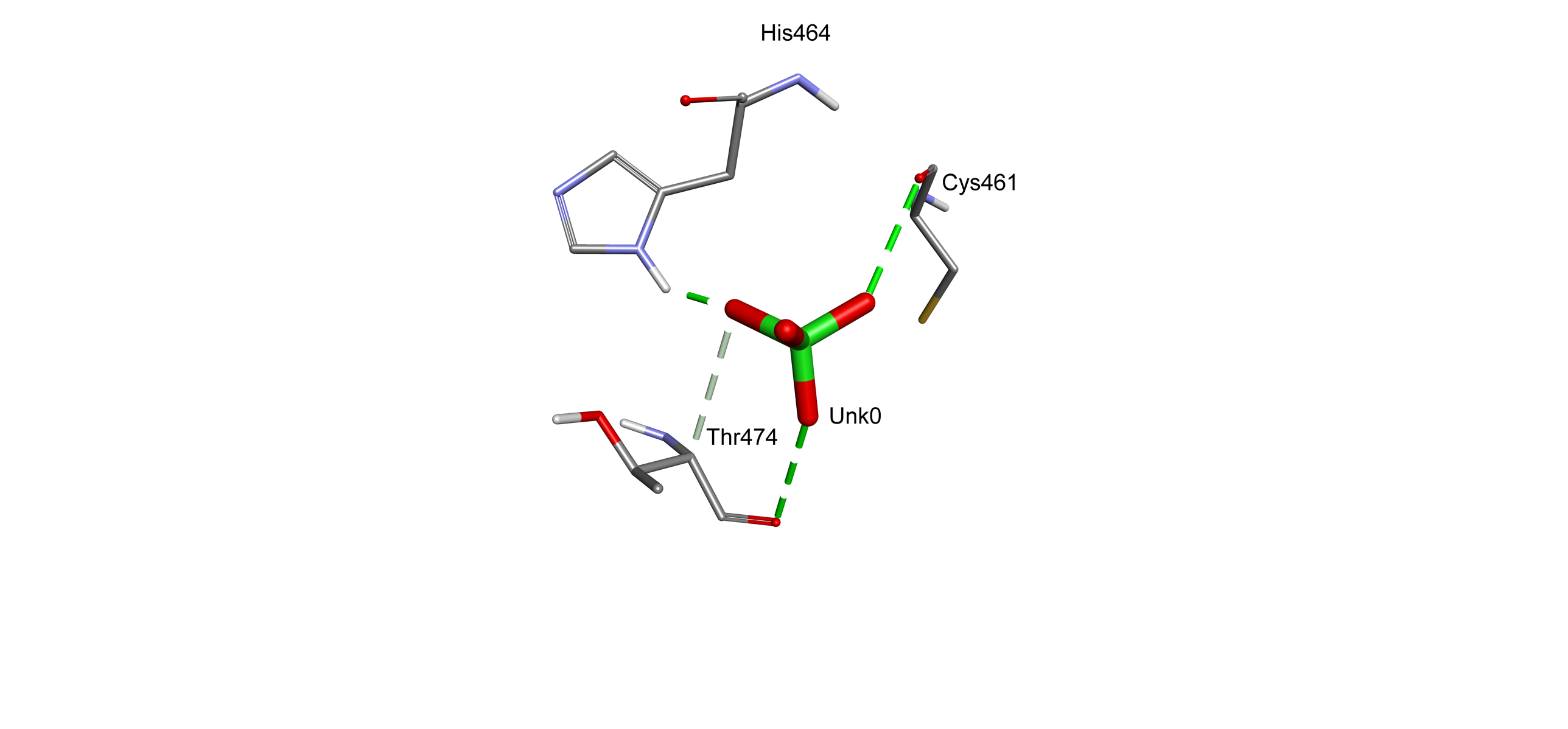


(B) Molecular docked model of HSA + ClO_4_^-^

**Figure S9.** Molecular docked model of HSA in presence of anions
